# PPAR-*γ* Agonists and Their Effects on IGF-I Receptor Signaling: Implications for Cancer

**DOI:** 10.1155/2009/830501

**Published:** 2009-07-07

**Authors:** A. Belfiore, M. Genua, R. Malaguarnera

**Affiliations:** ^1^Endocrinology Unit, Department of Clinical and Experimental Medicine, University of Catanzaro, 88100 Catanzaro, Italy; ^2^Endocrinology Unit, Department of Internal and Specialistic Medicine, Garibaldi-Nesima Hospital, University of Catania, 95122 Catania, Italy

## Abstract

It is now well established that the development and progression of a variety of human malignancies are associated with dysregulated activity of the insulin-like growth factor (IGF) system. In this regard, promising drugs have been developed to target the IGF-I receptor or its ligands. These therapies are limited by the development of insulin resistance and compensatory hyperinsulinemia, which in turn, may stimulate cancer growth. Novel therapeutic approaches are, therefore, required. Synthetic PPAR-*γ* agonists, such as thiazolidinediones (TZDs), are drugs universally used as antidiabetic agents in patients with type 2 diabetes. In addition of acting as insulin sensitizers, PPAR-*γ* agonists mediate in vitro and in vivo pleiotropic anticancer effects. At least some of these effects appear to be linked with the downregulation of the IGF system, which is induced by the cross-talk of PPAR-*γ* agonists with multiple components of the IGF system signaling. As hyperinsulinemia is an emerging cancer risk factor, the insulin lowering action of PPAR-*γ* agonists may be expected to be also beneficial to reduce cancer development and/or progression. In light of these evidences, TZDs or other PPAR-*γ* agonists may be exploited in those tumors “addicted” to the IGF signaling and/or in tumors occurring in hyperinsulinemic patients.

## 1. Introduction

Traditional anticancer therapies as chemotherapy and radiations are often unable to eradicate advanced cancers. Novel therapeutic modalities are, therefore, needed in the aim to lower the threshold for cancer cell death induced by traditional therapies.

The insulin-like growth factor (IGF) system has recently emerged as having a relevant role in cancer development and progression and in the resistance to drug-induced apoptosis. It is now well established that the IGF system is dysregulated/overactivated in a variety of human malignancies. Common mechanisms of dysregulation include autocrine and/or paracrine secretion of insulin-like growth factors (IGF-I and IGF-II) and overexpression of their cognate receptors (the IGF-I receptor, IGF-IR, and the closely related insulin receptor, IR). One viable anticancer strategy is, therefore, to target the various IGF system components that are dysregulated and that sustain increased constitutive IGFs' signaling in cancer cells.

Most anticancer strategies designed to curtail the IGF system dysregulation have been designed to target the IGF-IR [1–3]. In this regard, promising drugs have been developed, as small molecules with specific IGF-IR tyrosine kinase inhibiting activity and antiIGF-IR monoclonal antibodies that cause ligand binding inhibition and receptor downregulation and degradation. Other approaches have chosen IGF-I and/or IGF-II as targets. Some of these compounds have shown promising activity in preclinical studies and are now being evaluated in phase I and phase II clinical trials. The aberrant expression of the insulin receptor isoform A (IR-A) in malignant cells has also been advocated as a target. One limit of all these targeted therapies is the occurrence of insulin resistance and compensatory hyperinsulinemia caused by either direct impairment of the IR function or by growth hormone (GH) increase in response to the reduced IGFs signaling [4, 5].

Unfortunately, several epidemiological studies have shown that a high level of circulating insulin (hyperinsulinemia) is associated with an increased risk for a number of malignancies [6]. Moreover, hyperinsulinemia is very common in western societies because closely associated with obesity and type 2 diabetes [4, 7, 8].

Thiazolidinediones (TZDs) are synthetic PPARs agonists that are widely used as antidiabetic agents in patients with type 2 diabetes. These drugs ameliorate tissue sensitivity to insulin and, indirectly, cause a reduction of circulating insulin levels. Moreover, TZDs, as other PPAR-*γ* agonists, such as the prostanoid 15d-PGJ2 [9], induce a variety of favorable changes (growth arrest, apoptosis, and/or partial redifferentiation) in several malignancies, including liposarcoma, and cancers of the breast, colon, pancreas, and prostate [10–18].

We will herein review the available evidences indicating that these anticancer effects of PPAR-*γ* agonists are partially related to the downregulation of the IGF system activity at various levels. On the basis of these evidences, we suggest that TZDs or other PPAR-*γ* agonists may be a useful adjunct to the therapy of IGFs-driven malignancies.

## 2. The IGF System and Its Role in Cancer

### 2.1. Components of the IGF System

**Figure 1:**
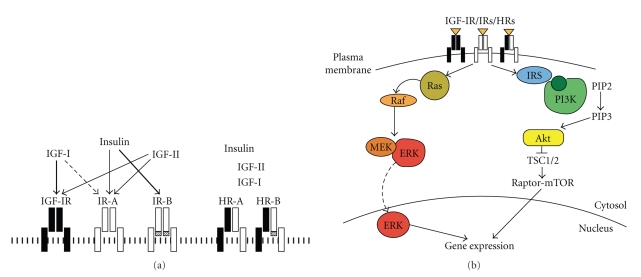
*The IGF system: receptors, ligands, and signaling pathways. * (a) * Schematic representation of the major receptors and ligands involved in the IGF system. * Insulin receptor isoforms (IR-A or IR-B) binds insulin with high affinity, while IGF-I receptor (IGF-IR) binds IGF-I and IGF-II (left). In cells expressing both IR and IGF-IR, IR hemireceptors may heterodimerize with IGF-IR hemireceptors, leading to the formation of hybrid IR/IGF-IRs (HRs), which bind IGF-I and IGF-II with high affinity and insulin with a much lower affinity (right). (b) *The IGF system signaling pathways*. Schematic representation of the two major signaling pathways involved in IGF system. IGF-I, IGF-II, and insulin bind to their cognate receptors, leading to the activation of the PI3K/Akt/mTOR pathway and of the Ras/Raf-1/MEK/ERK pathway.

The IGF system is composed by at least two closed related receptors, three ligands (insulin and insulin-like growth factors I and II) and six ligand-binding proteins (IGF-BPs) (Figure 1(a)) [19, 20]. The two receptors, the type I IGF receptor (IGF-IR) and the insulin receptor (IR) are tetrameric glycoproteins composed of two extracellular *α*- and two transmembrane *β*-subunits linked by disulfide bonds [21, 22]. The *α*-subunits contain the ligand binding site while the *β*-subunits contain a tyrosine kinase domain. These two receptors share more than 50% overall amino acid sequence homology and 84% homology in the tyrosine kinase domains. The transmembrane domain has a crucial role in recruiting intracellular mediators [23] through two conserved tyrosine residues.

The IR exists in two isoforms that differ for the inclusion (isoform B or IR-B) or the exclusion (isoform A or IR-A) of 12 aminoacid residues encoded by exon 11 [24, 25]. These two IR isoforms appear to have different ligand specificity and tissue distribution [26–29].

Because of the high sequence similarity between the IR and the IGF-IR [21, 22], an IR hemireceptor may assemble not only with a second IR hemireceptor but also with an IGF-IR hemireceptor, forming a hybrid IR/IGF-IR receptor (HR).

The signaling of these receptors regulates crucial functions of multicellular organisms, such as glucose metabolism and growth and life span in response to nutrients [30, 31]. 

IGF-I and IGF-II expressions were first analyzed in rodents where IGF-II gene is widespread expressed in prenatal period and diminishes dramatically after birth. In contrast, IGF-I levels are low during the prenatal period and increase significantly during puberty and adulthood. The overall picture of IGFs expression in rodents initially led to the associate IGF-II as a fetal growth factor and IGF-I as an adult growth factor. However, this expression pattern is not observed in humans, as both IGF-I and IGF-II are produced in human tissue during lifetime period.

The human IGF-I gene is located on chromosome 12 and has two promoter sites [20]. Mature IGF-I is a 7.7 kDa protein that has 62% homology with IGF-II in its amino acid sequence [20]. The human IGF-II is encoded by a 9-exons gene located on chromosome 11p15. Multiple transcripts are synthesized as a result of alternate promoter usage [32]. Promoter 1 is active only in adult liver, while P2-4 promoters are active in most fetal tissues. Activation of P3 and P4 promoters is common in cancer cells [33].

The biological activity of the IGF ligands is also modulated by a family of high-affinity IGF binding proteins (IGFBP 1–6). IGFBP-3 is the predominant binding protein expressed in serum, and most circulating IGF-I and IGF-II are bound in a ternary complex with IGFBP-3 and a third component, the acid-labile subunit (ALS). The IGFBPs rule IGF action by increasing the half-lives of circulating IGFs, by controlling their availability for receptor binding. IGFBPs-1–4 have similar affinities for IGF-I and IGF-II whereas IGFBP-5 and 6 bind IGF-II with a 10- and 100-fold higher affinity, respectively, than IGF-I [34–36].

A detailed description of the different components of the IGF system and of the complex signaling network activated by ligand binding to the IGF-IR and the IR is beyond the scope of this article and can be found elsewhere [37]. We will herein provide a brief overview of the major signaling pathways common to both receptors in order to describe the consequences of the IGF system dysregulation frequently observed in cancer and the way PPAR-*γ* agonists may affect these pathways.

### 2.2. The IGF System Signaling Pathways

Both IGF-I and insulin bind to the extracellular *α*-subunit of their cognate receptor and induce a conformational change that causes the activation of the receptor tyrosine-kinase and the autophosphorylation of tyrosine residues of the intracellular *β*-subunit at the level of the catalytic domain, the juxtamembrane domain and the C-terminus [38]. Tyrosine phosphorylation causes the recruitment of several intracellular substrates which function as either docking proteins (IRS-1, IRS-2) or adaptors (SHC, Grb2) for other intracellular proteins that have specific recognition domains, termed Src-homology-2 (SH2) domains [39]. In turn these substrates bind and recruit other intracellular proteins (Figure 1(b)) [40].

IRS-1, which binds to phosphotyrosine residues in the juxtamembrane domain with its Phosphotyrosine Binding (PTB) domain, has 20–22 potential tyrosine phosphorylation sites surrounded by possible binding sites for SH2-proteins [39]. Among the relevant SH2 domain-containing proteins, the phosphatidylinositol-3 kinase (PI3K) [41] and the GTPase activating protein (GAP) of Ras [42] originate the two major signaling pathways, common to both the IR and the IGF-IR.

The PI3K binds to IRS proteins through its regulatory subunit (p85), which then recruits the PI3K catalytic subunit (p110) to the plasma membrane, where it phosphorylates phosphatidylinositol-(4,5)-bisphospate (PIP2) to phosphatidylinositol-(3,4,5)-trisphosphate (PIP3). PIP3, in turn, recruits the protein kinase Akt and allows its activation by the phosphoinositide-dependent kinase 1 (PDK1). Akt activation is crucial for the regulation of glucose metabolism but also for the regulation of cell size, proliferation and survival by regulating metabolic enzymes, such as glycogen synthase kinase 3 and 6-phosphofructo-2-kinase and apoptosis modulators, such as BAD. Akt also regulates mRNA translation through the raptor-mTOR pathway, which also has a central role in cell growth and metabolism [43, 44]. Activation of the raptor-mTOR complex requires the binding with Rheb (Ras homolog enriched in brain), a GTP-binding protein. Rheb binding to GTP is regulated by the TSC1/TSC2 heterodimeric complex that functions as a specific GTPase-activating protein (GAP). 

Akt phosphorylates and inactivates TSC2, thus increasing the amount of active GTP-Rheb that binds and activates the raptor-mTOR complex. Two main signaling pathways originate downstream the raptor-mTOR complex: the p70S6 kinase (p70S6K) and the eIF4E-binding protein1 (4E-BP1) pathway. The p70S6K is a serine/threonine protein kinase, which regulates the synthesis of factors involved in the protein synthesis machinery, including the ribosomal S6 protein, and the translational regulators eukaryotic translation elongation factor 2 (eEF2) kinase and eukaryotic translation initiation factor 4B (eIF-4B).

The 4E-BP1 pathway regulates the activity of eukaryotic translation initiation factor 4G (eIF-4G) protein, which is involved in cap-dependent mRNA translation. After phosphorylation by raptor-mTOR, 4E-BP1 releases the eukaryotic translation initiation factor 4E (eIF-4E) allowing it to interact with eIF-4G, thus activating cap-dependent mRNA translation [45]. 

Akt also directly regulates gene transcription by promoting inactivation of transcription factors of the forkhead box “Other” (FoxO) family. In the basal state FoxO proteins are located in the nucleus where they activate the transcription of molecules relevant to metabolism, apoptosis promotion and cell cycle inhibition [46–49]. Following phosphorylation by Akt, FoxO factors are bound by 14-3-3 proteins and sequestered in the cytoplasm [50–52]. 

The PI3K/Akt pathway is negatively regulated by the lipid phosphatase PTEN, which dephosphorylates PIP3 [53]. PTEN activity is often reduced in cancer by mutations, underlying the key role of Akt pathway in cancer biology [54]. 

The second major signaling pathway downstream the IR and the IGF-IR involves Ras, a GTP-binding protein that cycles from the active (GTP-bound) to the inactive (GDP-bound) form [42]. Ras is activated by Ras guanine nucleotide exchange factor mSOS, which binds the SH3 domain of the adaptor Grb2. Grb2 couples the Ras/mSOS complex to the IR or IGF-IR by binding both to phosphorylated Shc and IRS proteins. Both IRS proteins and Shc compete for the same phosphosites on the receptor transmembrane domain [55]. After activation, Ras recruits to the membrane and activates the serine/threonine kinase Raf, which phosphorylates the dual specificity kinase MEK that in turn phosphorylates and activates ERK1/2 kinases. Inactive ERK1/2 is mainly located in the cytoplasm where it forms a MEK/ERK heterodimer [56]. After activation, ERK1/2 translocates to the nucleus where it phosphorylates a number of substrates involved in the transcription activation of several genes [42, 57, 58]. Moreover, activated ERK1/2 phosphorylates also numerous substrates in the cytoplasmic compartment implicated in cell growth and survival [58], such as p90 ribosomal S6 kinases (RSKs). RSKs inactivate proapoptotic proteins and contribute to positively regulate mRNA translation through p70S6K and 4EBP1.

### 2.3. The IGF System in Cancer Development

Both epidemiological and experimental evidences have suggested a crucial role of the IGF system in cancer development and progression. Elevated plasma concentrations of IGF-I have been linked to an increased risk for several malignancies [59–62]. In particular, subjects with serum IGF-I levels in the upper quartile of the normal range have been shown to be at increased risk of premenopausal breast cancer and other cancers, such as prostate, lung, colorectal, endometrial and bladder as compared to subjects who have values in the lower quartile [34, 59, 60, 62, 63]. Evidences supporting a correlation between IGF-I levels and cancer risk arise also from acromegalic patients. Indeed, epidemiological studies suggest that high GH and IGF-I levels in acromegaly are associated with an increased incidence of various malignancies, including colon cancer [64].

More recently, it has also become evident that high circulating levels of insulin are an important factor of cancer promotion. Insulin resistance and associated compensatory hyperinsulinemia are common features of obesity and type 2 diabetes. In western countries obesity now occurs in approximately 30% of the general population and type 2 diabetes in 5-6%. The prevalence of both these disorders increases with age. Both obesity and type 2 diabetes are associated with an increased risk for many forms of cancer, including cancer of the breast, colon, liver, pancreas, kidney and others [7, 8, 65]. Actually, hyperinsulinemia seems to be the major link between these disorders and cancer [4] and is also associated with a poor cancer prognosis [66]. 

Moreover, a variety of in vitro and in vivo studies have provided evidences of a complex dysregulation of the IGF system in cancer cells and have elucidated a few mechanisms by which this dysregulation may result in cancer promotion. The IR and the IGF-IR do not appear mutated in cancer but are often overexpressed [67–69]. With regard to IR, it is worth mentioning that only one of the two IR isoforms, the A isoform or IR-A, is overexpressed in cancer [70]. Interestingly, IR-A is a high affinity receptor not only for insulin but also for IGF-II [71]. As a further layer of complexity, cancer cells express high levels of IR/IGF-IR hybrid receptors as a consequence of IGF-IR and IR overexpression [72]. 

Moreover, cancer cells often show abnormal autocrine/paracrine production of both IGF-I and IGF-II, potent mitogens that bind both the IGF-IR and the IR-A [73, 74]. Several studies have demonstrated that forced expression of IGF-I and IGF-II in transgenic animal is associated with accelerated cancer development [75–78]. 

IGF system dysregulation/overactivation resulting from receptor and ligand abnormal expression may favor cancer progression by various mechanisms that depend on the constitutive activation of the two main branches of intracellular signaling, the PI3K/mTOR and the ERK1/2 signaling pathways. Interestingly, PTEN, a lipid phosphatase important for restraining the PI3K pathway, is often inhibited in human carcinomas and may contribute to the abnormal signaling of the IGF system [79]. This abnormal signaling leads to various effects, including upregulation of cyclin D1 and cyclin-dependent kinase 4 (CDK4), retinoblastoma (RB) phosphorylation and activation of cyclin E [80, 81] and enhanced expression of matrix metalloproteases (MMPs) [82].

This eventually results in the promotion of cell proliferation and survival and enhanced cell migration [83]. Finally, the IGF-IR has also a crucial permissive role for anchorage-independent cell growth, which is strictly associated with the malignant phenotype [84, 85]. In accordance with these findings, IGF-IR knocking-out prevents cell transformation by several oncogenes [86, 87]. IR has similar although not identical effects as the IGF-IR. IR overexpression is also associated with a ligand-dependent transformed phenotype [88, 89] and is able to stimulate growth [90, 91] and chemotaxis [92, 93]. Finally, IGF system dysregulation in cancer induces resistance to radiation and various other targeted and nontargeted cancer therapies and has stimulated the development of therapeutical agents able to target IGF-IR signaling in cancer [94, 95].

## 3. Anticancer Effects of PPAR-*γ* Agonists

### 3.1. PPARs Family, Expression and Ligands

PPARs *(peroxisome proliferator activated receptors) * belong to the nuclear receptor family, and recently have emerged as transcription factors that regulate diverse aspects of metabolism [96].

**Figure 2:**
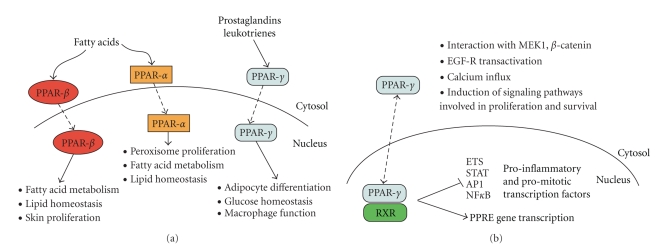
*Components and signaling pathways of the PPAR system. * (a) *Schematic representation of components of the PPAR system. * PPARs act as ligand-activated transcription factors that are responsive to the lipid status of the cell. The physiological ligands for these nuclear receptors are typically unsaturated fatty acids (FFAs) and their eicosanoid products. PPARs regulate the expression of genes that encode proteins involved with lipid metabolism (oxidation), leukotriene degradation, energy balance, eicosanoid signaling, cell differentiation and tumorigenesis. PPARs are differentially expressed in the various tissues. PPAR-*α* is highly expressed in liver, kidney, heart, brown adipose tissue, and the intestine, whereas PPAR-*γ* is found in adipose tissue, small intestine, and lymphatic tissues. PPAR-*β* is ubiquitous. (b) *PPAR-*γ* genomic versus nongenomic actions*. PPAR-*γ* belongs to the class of nuclear receptors, with a typical modular structure composed by at least an N-terminal transactivation domain and a DNA binding domain (DBD). Upon ligand binding, a conformational change leads to the release of corepressors (NCoRs), recruitment of coactivators (NCoAs), heterodimerization and transactivation of PPRE-related promoters. This genomic function of PPAR-*γ* controls immune response, as well as lipid and glucose metabolism. Nuclear PPAR-*γ* exerts also a negative cross-talk towards major proinflammatory and promitotic transcription factors. Cytoplasmic PPAR-*γ* by interacting with proteins (MEK1, *β*-catenin) and activating transmembrane proteinases, elicits rapid and transient nongenomic effects that modulate EGF-R transactivation, calcium influx, and PI3K/Akt, IKK/NF*κ*B and MAPKs signaling pathways.

To day, three PPARs have been identified—PPAR-*α*, PPAR-*β* (known also as *δ*) and PPAR-*γ* (Figure 2(a)). PPAR-*α* is expressed in liver, hearth, muscle and vascular wall whereas PPAR-*β*/*δ* is mainly expressed in skin, brain and adipose tissue [9, 97]. PPARs regulate gene expression upon ligand binding that drives heterodimerization with the retinoid X receptor (RXR) and subsequent binding to specific response elements located in the promoter regions of target genes (Figure 2(a)).

Due to his abundance in adipose tissue, pancreatic *β*-cells, vascular endothelium and also in macrophages, PPAR-*γ* is widely studied. Moreover, PPAR-*γ* is the molecular target for the synthetic thiazolidinediones (TZDs), clinically used as insulin sensitizers in patients with type II diabetes. Seven PPAR-*γ* mRNAs have been identified. PPAR-*γ*-1 and -2 are expressed mostly in adipose tissue and large intestine; lower levels are found in kidney, liver and small intestine. PPAR-*γ*-3 is found in adipose tissue and large intestine, PPAR-*γ*-4 and -5 are expressed only in macrophages, whereas PPAR-*γ*-6 and -7 are found in adipose tissue [98].

The molecule of PPAR-*γ* consists of an N-terminal domain (also called A/B domain), which is responsible for ligand-independent transcriptional regulation. The DNA-binding domain (or C) contains two zinc-finger-like and an *α*-helical DNA binding motifs. Through the C domain, PPAR-*γ *recognizes PPRE (Peroxisome Proliferators Response Element) sequences in the regulated promoter regions. In the C-terminal is present the ligand-binding domain that potentiates the PPAR-*γ* ability to dimerize with RXR and recruit coactivators, such as Steroid Receptor Coactivator 1 (SRC1), Peroxisome Proliferator Receptor-*γ* Coactivator 1*α* (PGC-1*α*), TRAP220/PPAR*γ* binding protein (PBP), and p300/CREB Binding Protein (CBP) (Figure 2(b)) [99–101]. The activation of PPAR-*γ* by its ligands leads concomitantly to its ubiquitination and eventually to proteasome degradation.

The ligand-binding pocket of PPARs is larger than that of other nuclear receptors and allows the binding for a variety of ligands. In addition to TZDs (exogenous agonists), these ligands include peroxisome proliferators such as nanenopin, clofibric acid and warfarin [102, 103]. Although endogenous ligands are yet not well defined, it has been shown that mono- and poly-unsaturated fatty acids and arachidonic acid metabolites may activate PPARs [104, 105].

### 3.2. PPAR-*γ* and Cancer: Mechanisms Involving Differentiation, Proliferation and Apoptosis

TZDs have been used for the treatment of hyperglicaemia in type II diabetes since 1997. Troglitazone was the first of this class of drugs introduced into clinical practice, but it was withdrawn because of liver toxicity. Currently, pioglitazone and rosiglitazone are the only compounds licensed for the treatment of patients with type II diabetes.

Recently, the interest in TZDs as potential anticancer agents has been raised by observations that TZDs and other PPAR-*γ* agonists possess PPAR-*γ* dependent and independent antitumor effects [106]. Thus, TZDs in combination with chemotherapy drugs or other targeted therapies may represent a promising tool in the treatment of malignancies [106].

The molecular basis for the antitumor action of PPAR-*γ* agonists remains incompletely elucidated. Numerous studies support the notion that PPAR-*γ* activation induces apoptosis and thus exerts anticancer effects [107]. For instance, in lung cancer cells troglitazone triggered apoptotic response in PPAR-*γ* and ERK1/2 dependent manner [108]. Troglitazone treatment reduced the antiapoptotic protein, Bcl-2, and caused nuclear accumulation and colocalization of PPAR-*γ* and ERK1/2 [108]. Moreover, in anaplastic thyroid cancer, rosiglitazone-induced apoptosis was associated with a decrease of Bcl-X_L_ expression and caspase-3 and -7 activation [109]. 

In addition to apoptosis, PPAR-*γ* activation may reduce tumor development by halting cancer cell proliferation [110]. One well-know mechanism for suppressing proliferation involves cell cycle arrest. Cyclins are cell cycle regulators that specifically activate cyclin-dependent kinases (CDKs). Due to their role in cell cycle control, cyclins are potential oncogenes: in fact, cyclin D1 is overexpressed or amplified in several human cancers [111, 112].

Exposure to TZDs for 24 hours may cause G_0_/G_1_ cell cycle arrest [113, 114]. TZD treatment not only decreased protein levels of cyclin D1, but also reduced proliferating cell nuclear factors such as pRb and Cdk4, and increased the cyclin-dependent kinase inhibitors p21 and p27, in a time dependent manner [114]. Again, in thyroid anaplastic cancer rosiglitazone induced growth arrest increasing cyclin-dependent kinase inhibitors p21 and p27, reducing cyclin D1 expression as well as activating Rb protein [109].

Another mechanism by which PPAR-*γ* activation may act as tumor suppressor is through the promotion of cellular differentiation. In cultured breast cancer, PPAR-*γ* ligands caused extensive lipid accumulation and changes in epithelial gene expression associated with a more differentiated, less malignant state [115]. In lung cancer cells, ciglitazone induced differentiation [116] and in thyroid cancer cells, rosiglitazione induced the expression of thyroid-specific differentiation markers suggesting a partial reversion of the epithelial mesenchymal transition [109].

The mechanism of TZD-induced differentiation has also been well studied in adipocytes and involves the interaction with the Wnt pathway. It has been shown that PPAR-*γ* and GSK3*β* interact through CAAT/enhancer binding proteins (C/EBP). GSK3*β*-induced C/EBP*β* activates PPAR-*γ*, that in turn activates the differentiating factor C/EBP*α* leading to the production of adiponectin and completion of adipocyte differentiation [117, 118].

Moreover, PPAR-*γ* and *β*-catenin expression seems to be correlated [119]. Direct interactions among PPAR-*γ*, RXR and *β*-catenin have been found recently in human kidney embryonic HEK293 cells and in human metastatic prostate cancer LNCaP cells [120, 121]. PPAR-*γ* can function to suppress Wnt signaling by targeting phosphorylated *β*-catenin to proteasome. In agreement with these findings, in 3T3-L1 adipocytes, *β*-catenin mRNA and protein levels were decreased by PPAR-*γ* activation [122]. The above data imply a positive feedback mechanism involving GSK3*β*, PPAR-*γ* and *β*-catenin that amplifies signals for differentiation and inhibits signals for proliferation in both adipocytes and cancer cells.

Another mechanism by which PPAR-*γ* agonists may mediate suppression of tumor initiation and progression is the inhibition of angiogenesis and the downregulation of tumor microenvironment inflammation [123, 124]. The PPAR-*γ* antiinflammatory actions are of relevance to the treatment of atherosclerosis as well as cardiovascular disease [125]. However, given the association of chronic inflammation with cancer risk, they may also prove important for the treatment and prevention of cancer. 

In summary, PPAR-*γ* agonists may act as negative regulators of cancer growth and progression, by multiple mechanisms. Some of these effects may be related to the direct and/or indirect interaction of PPAR-*γ* agonists with the IGF system. We will herein review the molecular mechanisms by which PPAR-*γ* agonists cross-talk with the IGF-system signaling and may affect cancer biology.

## 4. Cross-Talk between PPAR-*γ* Agonists and IGF System Signaling

**Figure 3:**
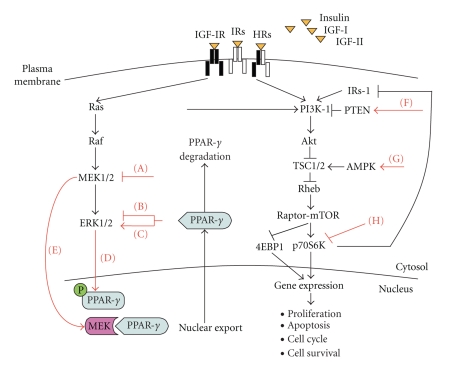
*Cross-Talk between PPAR-* *γ* *and IGF system downstream signaling pathways*. The points of interaction between PPAR-*γ* and MAPK/PI3K pathways occur at different levels and are indicated as arrows (activation) or bars (inhibition). In some cell contexts PPAR-*γ* ligands reduce MEK1/2 protein expression (A) and inhibit ERK1/2 phosphorylation (B). However, in other cell systems, PPAR-*γ* agonists may activate ERK1/2 (C). Alternative mechanisms of interaction between PPAR-*γ* and MAPK/PI3K pathways include (D) ERK-mediated PPAR-*γ* phosphorylation at Ser84/114; (E) MEK1/2-dependent PPAR-*γ* nuclear export followed by PPAR-*γ* degradation; (F) PTEN upregulation; (G) mTOR downregulation via activation of AMPK; (H) inhibition of p70S6K phosphorylation.

Conflicting evidence has led to confusion about whether PPAR-*γ* exerts an inhibitory or stimulatory effect on tumorigenesis (Table 1). It is generally considered as a tumor-suppressor, yet has been suggested to exacerbate the growth of certain tumors. The janus-face (anti- versus protumor function) of PPAR-*γ* and its ligands, can be explained by considering the interaction with IGF system and its downstream signaling pathways as MAPK, PI3K and mTOR (Figure 3). 

### 4.1. PPAR-*γ* and MAPK Cross-Talk: A Complex Issue Affecting IGF Signaling

As we have already mentioned in the previous section (the IGF system signaling pathways) the MAPK cascade transmits proliferative signals generated by cell surface receptors and cytoplasmic elements to the nucleus [152, 153].

The RTK-Ras/Raf/Mek/Erk pathway is overactivated in ~30% of human cancers [154] where it provides growth and survival signals. Growth factors can be overexpressed and receptor tyrosine kinases mutated or amplified, thereby stimulating constitutive receptor tyrosine kinases signaling [155]. One of the upstream regulators of MAPK pathway is the IGF signaling axis. The binding of IGF-I or IGF-II to the IGF-IR initiates conformational changes that triggers autophosphorylation and subsequent activation of Ras/Raf/Mek/Erk cascade. Thus, the IGF system deregulation through either (i) IGF-IR overexpression or (ii) autocrine/paracrine production of IGF-I and IGF-II or (iii) autocrine IGF-II/IR-A loop activation, can induce constitutive stimulation of the MAPK cascade.

The IGF mediated MAPK activation, in turn, may modulate the genomic activity of PPAR-*γ* by the inhibition of its antiproliferative and prodifferentiating functions, thereby contributing to the tumor promoting role of IGF pathway.

The points of interactions between MAPK pathway and PPAR*γ* are various [156, 157] and include the following.

(1) Phosphorylation of a serine residue of PPAR-*γ* (and its cofactors) by ERK1/2, JNK and p38 MAPKs (Figure 3(D)) [158]. Phosphorylation of PPAR-*γ* by MAPKs is directed against Ser82/112 (mouse) or Ser84/114 (human) within a consensus MAPK-motif (PxSPP) [159, 160]. This MAPK-dependent mechanism, modulates the ability of PPAR-*γ* to form a heterodimer with RxR and bind PPRE, thus inducing gene transcription (genomic activity of PPAR-*γ*) [161]. The PPAR-*γ* conformational change, induced by phosphorylation, inhibits its ligand-binding affinity [162], decreases its binding to PRRE [163] and the recruitment of coactivator/corepressor complexes [164]. Moreover, PPAR-*γ* phosphorylation promotes its inactivation by poly-ubiquination, proteasomal degradation and sumoylation [165]. The suppression of PPAR-*γ* genomic activity, by MAPK-mediated phosphorylation, is in concordance with the antiproliferative, antiinflammatory and prodifferentiating role of PPAR-*γ* and is supported by several in vitro studies, conducted in normal as well as in cancer cells. For example, it has been shown that TFN*α* suppresses PPAR-*γ* function in hepatic stellate cells [126] as do the PGF2*α* in adipocytes [127], proinflammation lipid mediators HODEs in colon and prostate cancer cells [128], EGF and PDGF in preadipocytes (3T3-L1) and in murine fibroblasts (NIH-3T3) [129, 130]. In addition, other studies have revealed the poly-ubiquination and proteasomal degradation upon S82/112 phopshorylation of PPAR-*γ* as a mechanism of PPAR-*γ* inactivation in adipocytes [131, 132], breast [133] and colon cancer cells [134], after IFN*γ*, Her-2 and gastrin stimulation, respectively.

In summary, altogether, these in vitro evidences indicate that MAPK stimuli, through phosphorylation, inactivate PPAR-*γ*-dependent gene transcription in cancer cells and are in line with the antitumor promoting role of PPAR-*γ* [135, 136].

(2) Modulation of PPAR-*γ*'s nucleo-cytoplasmic compartmentalization induced by MEK1, upon cell stimulation with mitogens and PPAR-*γ*-ligands (Figure 3(E)). 

Burgermeister et al. [131] have demonstrated that MEK1 and PPAR-*γ* directly interact in resting cells and this association induces a nuclear export of PPAR-*γ* leading to reduction in its genomic functions. Cytosolic PPAR-*γ* then, is either subjected to proteosomal degradation or shunted to other cell compartments (ER/Golgi, cytoskeleton, lipid droplets, caveolae) to trigger extranuclear/nongenomic actions. Although the functions of exported PPAR-*γ* remain to be completely elucidated, it seems that this dynamic change in subcellular localization of PPAR-*γ* may influence the balance between its tumor suppressive and tumor promoting activities. Several studies have reported that PPAR-*γ* is mainly present in the nucleus of non neoplastic tissues, where it exerts classical genomic functions (antiinflammatory, antiproliferative, prodifferentiating). In contrast, it is expressed predominantly in the cytoplasm of tumor tissues of infiltrating breast carcinoma and salivary duct carcinoma, where it is associated with increased tumor aggressiveness and poor clinical prognosis of patients [137, 138]. A differential subcellular distribution of PPAR-*γ* has also been shown in human stomach cancer tissues, where the nucleo : cytoplasm ratio of PPAR-*γ* increased along with the progression of intestinal metaplasia to undifferentiated cancer [166]. Notably, in lung tumor samples, PPAR-*γ* expression was present in both the nucleus and the cytoplasm [167]. These results are indicative of a correlation between cell malignancy and PPAR-*γ* subcellular distribution.

In summary, both mechanisms of MAPK/PPAR-*γ* interaction described above (i.e., the MAPK-mediated PPAR-*γ* phosphorylation as well as the MEK-1 driven nucleo-cytoplasmic shuttle of PPAR-*γ*), may represent a way for enabling PPAR-*γ* nuclear genomic activity and support the protumor and antiapoptotic role of IGFI system and its downstream signal-transduction components Ras/Raf/MEK/ERK.

(3) Inhibition of MAPK pathway mediated by PPAR-*γ* ligands. 

In some cell contexts and tissue-specific systems, it has been shown that PPAR-*γ* agonists specifically abolished the phosphorylation of ERK1/2 in a dose and time dependent manner (Figure 3(B)) and also downregulated the protein expression of MEK1/2 (Figure 3(A)), inducing cell growth arrest and apoptosis [137, 168]. The inhibition of MAPK signals mediated by PPAR-*γ* ligands, is in line with their antiproliferating and proapoptotic functions and is in concordance with data from literature showing that PPAR-*γ* agonists block the biological actions of IGF-I.

The implication of these findings is that PPAR-*γ* agonists may represent a reasonable therapeutic approach in tumors where MAPK and/o IGF-I pathways are overactivated. However, several in vitro and in vivo studies have provided evidences indicating that PPAR-*γ* ligands may also trigger promitotic and prosurvival signals in certain cell contexts. It is well known that PPAR-*γ* agonists exhibit rapid (within 15 minutes) non genomic effects, including activation of signaling pathways as MAPK and PI3K/mTOR [139], upregulation of p21 and p27 [140, 169], induction of p53 [170], transient alterations in mitochondrial functions [171]. An early response of ERK1/2 in presence of PPAR-*γ* ligands has been reported in many studies and occurs under largely unknown mechanisms, which may or may not involve PPAR-*γ*. Takeda et al. [172] demonstrated that 15d-PGJ2 and thiazolidinediones activate the MEK/ERK pathway through PI3K pathway, leading to c-Fos mRNA expression and DNA synthesis [108, 141]. Ciglitazone and 15d-PGJ2 induce activation of MAPKs in primary astrocytes, preadipocytes, chondrocytes and liver epithelial cells through a PPAR-*γ* independent mechanism [142, 168]. Patel et al. [140] have reported that troglitazone activates both PI3K and MAPK signaling networks. Furthermore, in colon carcinoma cells, it has been found that tiazolidinediones, through ROS production and ERK activation, induce matrix metalloproteinase 2 (MMP2) and subsequently increase tumor cell invasion [142]. Yet, in human breast cancer cells MCF7, 15d-PGJ2 upregulates VEGF and induces PPAR-*γ*-independent ERK activation, thereby stimulating angiogenesis and proliferation [143]. This positive cooperation between PPAR-*γ* and components of MAPK cascade in promoting tumor initiation and progression may explain the absence of therapeutic benefits of TZDs in some cancer patients and adds significant complexity to the PPAR-*γ* functions in cancer biology. Furthermore, the nongenomic rapid activation of ERK signaling cascade, in presence of PPAR-*γ* ligands, can affect the PPAR-*γ* genomic action, via ERK1/2—mediated PPAR-*γ* phosphorylation or via MEK—dependent PPAR-*γ* nuclear export. This overlap between genomic and nongenomic effects of PPAR-*γ* ligands, may explain both pro- and antitumor actions of PPAR-*γ* agonists. However, nowadays, the mechanisms underlying cell decision for pro- versus antiproliferative responses upon PPAR-*γ* ligands stimulation, are unknown.

So far, PPAR-*γ* emerges as a context-specific tumor modulator, whose functions are modulated by PPAR-*γ*-independent effects of its ligands and by a synergic or antagonistic cooperation with IGF-I/MAPK cascades. It is possible to speculate that in tumors where PPAR-*γ* expression/activity is low and the MAPK signaling network is constitutively activated by various stimuli, including IGF dependent signals, PPAR-*γ* ligands exert antitumor and antiproliferating effects. In this condition, the combined use of kinase inhibitors and PPAR-*γ* agonists may be beneficial as anticancer therapeutic strategy. Alternatively, the interactions between PPAR-*γ* and other important survival pathways, such as PI3K/mTOR, may influence cell fate and determine whether pro- or antitumorigenic responses are induced. In light of these considerations, elucidating the non genomic pathways of PPAR-*γ* ligands and delineating the transduction signals underlying the cross-talk between PPAR-*γ* and other key survival signals is likely to yield new therapeutic targets for development of anticancer therapies.

### 4.2. Cross-Talk between PPAR-*γ* and PI3K

Besides the MAPK cascade, alternative antiapoptotic and prosurvival signal transduction pathways, such as PI3K and mTOR, have been identified for the IGF-IR signaling axis (for a detailed description see Section 2.2).

The points of interaction between PPARs family and PI3K pathway involve different components of IGF-I/PI3K signaling cascade.

For example, as shown in adrenocortical cancer cells (SW13 and H295R), PPAR-*γ* ligands can rapidly interfere with the Akt phosphorylation/activation, which mediates IGF-I stimulated proliferation [173]. These evidences support the antiIGF-I role of PPAR-*γ* agonists observed in a wide variety of tumor cancer cell lines and tissues. A possible mechanism for this antagonistic effect is the increase in PTEN expression (Figure 3(F)). In line with this hypothesis, we have found that in human anaplastic cancer cell lines, rosiglitazone, via PTEN upregulation, inhibited IGF-I mediated biological effects such as cell migration, survival and anchorage-independent growth [109]. Similar results have been reported in human hepatocarcinoma cell lines (BEL-7404 and Hep3B) [144, 145] as well as in colon cancer cells (Caco2) [146], in breast cancer cells (MCF-7) [146], in nonsmall cell lung carcinoma cells (H1792, H1838 and A549) [147, 148], and in pancreatic cancer cells (AsPC-1) [149]. However, the exact molecular mechanism behind PTEN induction, due to PPAR-*γ* agonists, has yet to be fully understood. Two hypotheses have been proposed to explain the interaction between PTEN and PPAR-*γ*: PPAR-*γ* directly regulates PTEN transcription and/or PPAR-*γ* regulates secondary unknown factors that, in turn, regulate PTEN [146, 174]. Furthermore, because PTEN expression can be regulated by interfering with its transcription activity or by inducing posttranscriptional modifications, it is also possible that PPAR-*γ *agonists induce PTEN overexpression by decreasing its degradation [175, 176]. Aside from the specific mechanisms involved, the regulation of PTEN levels by PPAR*γ* agonists, provides a powerful mechanism to shut down the basal or stimulated signals of PI3K cascade and could be exploited for future treatment of cancers in which PTEN is downregulated/lost and IGF-I-mediated signals are amplified.

The link between PPAR-*γ* and PI3K pathway involves also another component of PI3K signaling axis: the mTOR/p70S6K cascade. As we have previously discussed, mTOR pathway is coregulated by IGF-I-Akt signaling to ensure both a reasonable level of nutrients and a positive mitogen signal for cell growth and division. The IGF-I system regulates the mTOR axis through the phosphorylation and inhibition of the TSC1/2 complex by Akt, which in turn activates Rheb and mTOR. Stimulation of the IGF-IR activates the PI3K/Akt/mTOR cascade and triggers an mTOR-dependent decrease in IRS-1 expression and phosphorylation, leading to PI3K/Akt/mTOR inactivation [177, 178]. This feedback inhibition could paradoxically reduce the antitumor effect of mTOR inhibition by enhancing IGF-I signaling.

Thiazolidinediones have been recently shown to modulate and regulate the IGF-I/Akt/mTOR/p70S6K pathway. In endothelial cells, troglitazone has been found to decrease p70S6K phosphorylation (Figure 3(H)) and to inhibit protein biosynthesis via a protein phosphatase 2A (PP2A)-dependent mechanism, which do not involve neither mTOR nor PPAR*γ* [150] An alternative mechanism through which PPAR*γ* agonists inhibits phosphorylation of p70S6K is the activation of AMPK (Figure 3(G)) [151]. AMPK is a multisubstrate enzyme induced by increase in AMP level during metabolic stress in response to exercise, hypoxia and fasting condition and it plays a major role in the regulation of energy control [179]. The phosphorylation of this fuel-sensing enzyme, negatively regulates mTOR by directly phosphorylating the TSC1/2 complex [180]. The mTOR inhibition induced by AMPK is in line with the existence of a link between AMPK and the growth inhibition of some cancer cells [179, 181, 182]. The activation of this enzyme induced by thiazolidinediones has been supported by several in vitro studies. As He et al. [151] have reported, PPAR*γ* agonists induced phosphorylation of AMPK in mouse skin keratinocytes most likely via a PPAR*γ*-indipendent mechanism, which subsequently suppresses IGF-I-induced cell proliferation by inhibiting mTOR activity and phosphorylation of p70S6K. Yet, Han and Roman [147] have found that rosiglitazone inhibits growth of nonsmall cell lung carcinoma cells via upregulation of AMPK, thereby downregulating the mTOR/p70S6K pathway through PPAR*γ*-dependent and/or PPAR*γ*-independent mechanisms. Although it is not well known how thiazolidinediones rapidly activate AMPK, the subsequent inhibition of mTOR signaling pathway can be considered a mechanism by which thiazolidinediones exert an antiIGF-I function as well as an insulin-sensitizing action.

As thiazolidinediones target both the IGF system and other crucial protumor signals downstream of IGF-IR, their use in monotherapy or in combination with the small-molecule TK inhibitors or with antibodies directed against the IGF-IR, may be effective to achieve the maximal therapeutic benefit and may even be useful to reduce the side effects associated with IGF-IR targeted therapy (insulin resistance, hyperglycaemia, hyperinsulinaemia, effect on growth and development of normal cells and tissues with high level of cellular turnover, neurotoxicity), thereby improving tolerability and/or efficacy. Furthermore, the use of PPAR*γ* agonists may represent a valid therapeutic tool to circumvent the resistance that some tumors develop to therapies targeting PI3K/mTOR signaling pathways.

### 4.3. PPAR-*γ* Agonists May Regulate IGFs Bioavailability

Hyperinsulinemia and insulin resistance, commonly observed in type 2 diabetes and others metabolic disorders, are associated with an increased risk of tumor development in several tissues. Compensatory activation of IGF-I signaling in insulin resistance states is believed to be responsible for this association [37, 183]. Thiazolidinediones are currently used to treat type 2 diabetes mellitus and, by ameliorating insulin resistance, have the potential to reduce IGF-I bioavailability in these patients. Furthermore, inhibiting the tumor promoting activity of IGF-I they may provide additional tumor preventive benefit to type 2 diabetes patients. The local availability of IGFs ligands is abnormally high in many cancers where they serve as endocrine, autocrine and paracrine regulators of survival and proliferating signals mediated by IGF-IR. The IGFs bioavailability, as well as the IGFs bioactivity, is modulated by IGF-I binding proteins (IGFBPs) and IGFP proteases. In general, IGFBPs limit IGFs access to IGF-IR, reducing the bioactivity of these growth factors. However, in some cell contexts, IGFBPs can increase rather than decrease IGF-mediated effects, by presenting and slowly releasing IGF-I for receptor interactions, while protecting the receptor from IGF-I induced downregulation [35]. This phenomenon has been shown for IGFBP1, IGFBP3 and IGFBP5 [37, 184]. Furthermore, IGFBPs (especially IGFBP3) may also regulate cell growth and apoptosis in IGF-I independent manner [185]. Alterations of individual components of IGF cascade (IGFs/IGF-IR/IGFBPs) may potentially contribute to cancer initiation and progression [186]. Several evidences have suggested that the effects of TZDs on cell proliferation and differentiation can be due to a modulation in IGFs/IGFBPs levels. Lecka-Czernik et al. [187] reported, that, in primary and transformed marrow stromal cells and in mouse liver cells, rosiglitazone, suppresses IGFI, IGFII and IGFBP-4 levels and reduces the expression of IGFI-R and IGFII-R, whereas no effect was observed in IGFBP-3 expression. These changes in IGF signaling effectors were evident within 72 hours and occurred at transcriptional as well as posttranscriptional levels. In addition, the modulation of the IGF system mediated by PPAR*γ* ligands, was associated with suppression in several cell cycle genes. These findings are in line with other reports demonstrating the antiproliferative effect and the antiIGF-I role of PPAR*γ* agonists [96, 188]. Although the PPAR*γ* mediated suppression of IGF-I levels could limit cell proliferation and induce apoptosis in specific cell context, the authors pointed out that the drop in circulating IGF-I may negatively impact the skeleton, by impairing osteoblastic differentiation and action [187]. These observations may have clinical significance and should be taken into account when TZDs are used in young patients that are still acquiring peak bone mineral density. However, further studies are needed to better understand the role of IGF-I in bone cell turnover. Interactions between PPAR*γ* and IGF-I signaling mediators have also been seen in human ovarian cells. In experiments carried out in vitro, TZDsincrease IGFBP-1 production by ovarian cells in the absence of insulin but enhanced insulin-mediated inhibition of IGFBP-1 production. The latter insulin sensitizing effect, could increase the bioavailability of IGFs, thereby enhancing the IGF-I mediated ovarian follicle proliferation and steroidogenesis [189]. These opposite effects on IGFBP-1 production (insulin-independent stimulation versus insulin-dependent inhibition) observed in vitro*, * contrast with in vivo data showing that TZDs increase of IGFBP-1 synthesis, presumably because they reduce hyperinsulinemia [190]. Stimulation of insulin receptor or IGF-IR as well as the modulation of circulating IGFBP-1 levels by TZDs may increase ovarian sensitivity to insulin and/or IGFs. The subsequent effect is the reduction of ovarian androgen production and the improvement of ovulatory function [191]. Although the role of TZDs in mediating ovarian function through IGFBP-1 synthesis modulation needs to be clarified, the interactions between PPAR*γ* agonists and IGF system may be important under both physiological (ovulation and steroidogenesis) and pathological conditions (polycystic ovary syndrome and syndromes of extreme insulin resistance). The effect of PPAR*γ* agonists on IGFBP-1 production has also been studied in HepG2 hepatoma cells. In these cells, troglitazone is able to increase IGFBP-1 mRNA expression and secretion in a PPAR*γ* independent manner [192]. IGFBP-1 mediates critical prosurvival signals in liver, although it has also been shown to inhibit the antiapoptotic IGFs functions. Nowadays, the exact role of IGFBP-1 production in liver cells upon PPAR*γ* agonists treatment, is yet not understood. The variety of the effects induced by TZDs on components of IGF-I system is still under investigation and may be due to the multiple and alternative signaling pathways they stimulate or inhibit. This adds significant complexity in the understanding of PPAR*γ* agonists functions in both physiological and pathological conditions.

### 4.4. PPAR-*γ* Agonists: In Vivo Studies

The role of PPAR-*γ* as a key regulator of metabolism is mainly mediated through its transcription factor activity in adipose tissue, muscle and liver. Studies in knock-out mice have elucidated this role and have demonstrated that insulin sensitization, through PPAR-*γ* action, involves adipose tissue maintenance [193–195].

The activated receptor works in a number of ways to achieve these effects, including alteration of the expression of genes involved in lipid metabolism, free fatty acid (FFA) uptake and storage in adipose tissue [196, 197]. Moreover, PPAR-*γ* activation by TZDs has been shown to reduce the amount of circulating FFA via adipocyte differentiation and apoptosis [198]: the number of small adipocytes, able to accumulate FFA, increases at the expense of hypertrophied ones that release FFA. As consequence, adipose tissue mass is increased, so other insulin-sensitive tissues are in an advantaged position. Glucose metabolism by liver and muscle is improved and *β*-cell apoptosis decreased, thereby increasing insulin secretion in type II diabetic patients. Another important effect of PPAR-*γ* activation is connected with increased adiponectin production from adipose tissue [196, 199]. Adiponectin significantly increases fatty acid oxidation in human skeletal muscle via activation of AMP-activated protein kinase, again with increased glucose uptake [199].

### 4.5. Effects on Insulin Serum Level and Circulating IGF-I

TZDs are useful agents in the treatment of hyperglycaemia, acting as insulin sensitizers and enhancing glucose uptake via their interactions with PPAR-*γ* receptors. As already mentioned, in vivo experiments in diabetic mice confirm that treatment with PPAR-*γ* agonists resulted in significantly improved glycaemia and increase in circulating adiponectin levels [200, 201].

Clinical trials have shown that TZDs lower fasting and postprandial glucose and it has been demonstrated that pioglitazone and rosiglitazone at maximal doses can lower glycosilated haemoglobin by 1–1.5%, on average [9]. Moreover, it has been shown that treatment with rosiglitazone slowed progression to monotherapy failure more effectively than metformin or glyburide. Rosiglitazone was also shown to slow the rate of loss of *β*-cell function and improve insulin sensitivity to a greater extent than the other two drugs [202].

Despite the growing amount of clinical data supporting the role of PPAR-*γ* agonists as insulin-sensitizers, only a small number of in vivo studies is available regarding the effect of PPAR-*γ* ligands on IGF-I serum level.

Nonetheless, current studies provide some essential and encouraging information. Lecka-Czernik et al. [187] have recently focused their attention on PPAR-*γ* role in the acquisition and maintenance of bone mass. In yellow obese agouti mice, rosiglitazone treatment for 8 week lowered serum IGF-I and reduced IGF-I transcript in liver and peripheral fat. Similarly, Ackert-Bicknell et al. [203] have recently analyzed strain specific effects of rosiglitazone on bone mass, body composition and serum IGF-I levels. They found that in the C3H strain, rosiglitazone affects adiposity and decreased circulating IGF-I levels.

## 5. Concluding Remarks

It is now widely accepted that IGF system dysregulation plays an important role in the development of common human malignancies. Cancer cells often overexpress IGF-IR and IR-A. Moreover, signaling downstream of these receptors is frequently constitutively activated by autocrine/paracrine production of IGF-I and/or IGF-II.

In addition, hyperinsulinemia, an important feature associated with the insulin resistance of obesity and type 2 diabetes may stimulate IGF-IR and IR-A in cancer cells and play a key role in cancer promotion [204–207]. Circulating levels of IGF-I in the upper quartile of the normal range may also promote cancer, especially at critical ages such as childhood and adolescence [36]. 

The pleiotropic effects of PPAR-*γ* agonists have several potential beneficial effects in cancer therapy. Not only PPAR-*γ* agonists downregulate both the PI3K and the Ras pathway, which are the two main signaling pathways downstream receptors of the IGF system, but also they ameliorate insulin resistance and lower circulating levels of insulin and free IGF-I. One may hypothesize that the janus-face (anti- versus protumor function) of PPAR-*γ* and its ligands can be explained by the interaction with IGF system and that anticancer effects are mainly to be expected in those tumors “addicted” to the enhanced IGFs signaling. Only scanty data are currently available regarding the antitumor effect of PPAR-*γ* agonists in the clinical setting [17, 106, 108, 208] and more work is needed in this field. Some disappointing results in clinical studies may be explained by the inclusion of cancers in far advanced stages. Moreover, in future trials we probably need to include tumors characterized by IGF system overactivation and/or tumors occurring in hyperinsulinemic patients. Finally, in vitro and in vivo studies have demonstrated that combination therapies, with PPAR-*γ* agonists together with other anticancer drugs, including drugs targeting the IGF system, may provide benefit for the treatment of certain human cancers. The most promising drugs able to potentiate the antiproliferative effect of PPAR-*γ* agonists are platinum-based chemotherapics, COX-2 inhibitors, paclitaxel, EGFR tyrosine kinase inhibitors, mTOR antagonists and IGF-IR inhibitors [148, 209–212]. Although combination therapies using these agents have been suggested to be effective in terms of antitumor activity and prevention of drug resistance, large randomized clinical trials are required for further evaluation and optimization.

## Figures and Tables

**Table 1:** Mechanisms underlying PPAR-*γ* pro- and antitumorigenic effects.

Protumorigenic effects	References
(1) Inhibition of PPAR-*γ* genomic functions by MAPK	[108, 126–143]
(2) PPAR-*γ* nongenomic functions (MAPK activation, MMP2, and VEGF production)	

Antitumorigenic effects	References

(1) PPAR-*γ* genomic effects	[109, 144–151]
(2) Induction of PTEN expression	
(3) Inhibition of p70S6K	
